# Temporal patterns of gene expression associated with tuberous root formation and development in sweetpotato (*Ipomoea batatas*)

**DOI:** 10.1186/s12870-015-0567-5

**Published:** 2015-07-16

**Authors:** Zhangying Wang, Boping Fang, Xinliang Chen, Minghuan Liao, Jingyi Chen, Xiongjian Zhang, Lifei Huang, Zhongxia Luo, Zhufang Yao, Yujun Li

**Affiliations:** Guangdong Provincial Key Laboratory of Crops Genetics and Improvement, Crops Research Institute, Guangdong Academy of Agricultural Sciences, Guangzhou, 510640 China

**Keywords:** Sweetpotato, Tuberous root, Transcriptome, Expression patterns

## Abstract

**Background:**

The tuberous root of sweetpotato is undisputedly an important organ from agronomic and biological perspectives. Little is known regarding the regulatory networks programming tuberous root formation and development.

**Results:**

Here, as a first step toward understanding these networks, we analyzed and characterized the genome-wide transcriptional profiling and dynamics of sweetpotato root in seven distinct developmental stages using a customized microarray containing 39,724 genes. Analysis of these genes identified temporal programs of gene expression, including hundreds of transcription factor (TF) genes. We found that most genes active in roots were shared across all developmental stages, although significant quantitative changes in gene abundance were observed for 5,368 (including 435 TFs) genes. Clustering analysis of these differentially expressed genes pointed out six distinct expression patterns during root development. Gene Ontology (GO) and Kyoto Encyclopedia of Genes and Genomes (KEGG) enrichment analysis revealed that genes involved in different processes were enriched at specific stages of root development. In contrast with the large number of shared expressed genes in root development, each stage or period of root development has only a small number of specific genes. In total, 712 (including 27 TFs) and 1,840 (including 115 TFs) genes were identified as root-stage and root-period specific, respectively at the level of microarray. Several of the specific TF genes are known regulators of root development, including DA1-related protein, SHORT-ROOT and BEL1-like. The remaining TFs with unknown roles would also play critical regulatory roles during sweetpotato tuberous root formation and development.

**Conclusions:**

The results generated in this study provided spatiotemporal patterns of root gene expression in support of future efforts for understanding the underlying molecular mechanism that control sweetpotato yield and quality.

**Electronic supplementary material:**

The online version of this article (doi:10.1186/s12870-015-0567-5) contains supplementary material, which is available to authorized users.

## Background

Sweetpotato (*Ipomoea batatas*), one of the most important food crops in the world, is mainly cultivated for its underground tuberous roots, which are rich in starch and other nutrients. Due to its wide adaptability, high yield, multiple uses and easy management, sweetpotato is grown around the world, especially in Asia and Africa. According to the Food and Agriculture Organization (FAO) statistics, world production of sweetpotato in 2010 was about 108 million tons, and the majority came from China, with a production of around 81 million tons from about 3.7 million hectares [1]. Furthermore, the sweetpotato tuberous root, involved in carbohydrate storage and vegetative propagation, is also a unique organ, which has the value of biological research for organogenesis and evolution. Therefore, understanding the processes regulating the tuberous root formation and development is of particular importance [2].

The formation of tuberous root depends mainly on two biological processes. Firstly, the primary cambium develops between the protophloem and protoxylem, and lignification of the stele is suppressed. Then later root thickening growth is primarily due to active cell division of the secondary meristems in the xylem [3–5]. Both processes have been shown to be affected by extrinsic environmental cues, including soil temperature, humidity, light intensity, photoperiod, carbon dioxide and nutrient status [6–12], and intrinsic hormone factors. The involvement of several plant hormone, including cytokinin, auxin, JA and ABA, in the formation and thickening growth of tuberous roots has been investigated [13–19]. These results lead to the hypothesis that these hormones possibly have different roles in the initiation and thickening processes of tuberous roots. To date, however, the distinct role of each hormone has not been directly elucidated.

Over recent years, considerable progress has been made in the isolation and characterization of genes associated with tuberous root formation. Using simplified differential display analysis, 10 genes were identified as being developmentally regulated, and the expression of sweetpotato class I *knotted1*-like homeobox genes in the storage roots was further confirmed [20, 21]. You *et al.* constructed a cDNA library with early stage storage roots and identified 22 differentially expressed genes in early storage root and fibrous root [22]. Noh *et al.* isolated a cDNA of a MADS-box protein (*SRD1*) from the same cDNA library and demonstrated that *SRD1* played a role in the formation of storage roots by activating the proliferation of cambium and metaxylem cells to induce the initial thickening growth of storage roots in an auxin-dependent manner [23]. Ku *et al.* [24] isolated *IbMADS1* from sweetpotato using cDNA-AFLP and analyzed its functional role in tuberous root initiation. However, the tuberous root formation and development of sweetpotato are complex biological processes involving morphogenesis as well as dry matter accumulation. The traditional approaches are not sufficient for elucidating the molecular mechanisms controlling the traits of interest. With the recently developed next generation sequencing (NGS) technology, large amount of transcribed sequences of sweetpotato have been generated and are available for systematic survey of the genes crucial for these important processes [2, 25–28]. Tao *et al.* identified differentially expressed transcripts in different tissues and at various developmental stages by using Illumina digital gene expression (DGE) tag profiling [26]. Firon *et al.* compared the expression profiles of initiating storage roots and fibrous roots using NGS platforms, and highlighted the down-regulation of lignin biosynthesis and up-regulation of starch biosynthesis at an early stage of storage root formation [28].

To further increase our understanding of the tuberous root formation and development, a whole transcriptome analysis of gene expression during these processes is needed. In this study, we investigated gene expression variations of sweetpotato root at seven different developmental stages by using a customized 60-mer oligonucleotide microarray. The primary objective of this study was to characterize global transcriptome expression patterns during the tuberous root formation and development, and to identify important candidate functional genes and key transcriptional regulators required for these processes.

## Results

### Sweetpotato unigene assembly, microarray design and gene annotation

An oligonucleotide microarray containing 39, 724 unique genes was created based on a large EST collection from publicly available database and in-house sequences (for further details, see Materials and methods). In this study, a total of 181,615 ESTs from a wide variety of sweetpotato tissues at various developmental stages or under different treatments were used as raw data for probe design. To eliminate redundant sequences and improve the sequence quality, the TIGR Gene Indices Clustering Tools (TGICL) [29] was used to obtain consensus sequences from overlapping clusters of ESTs. Assembly criteria included a 50 bp minimum match, 95 % minimum identity in the overlap region and 20 bp maximum unmatched overhangs. After assembling, a total of 87,492 tentative unique ESTs (hereafter referred to as "genes") including 28,885 contigs and 58,607 singletons were generated. Based on these genes, a NimbleGen 4 × 72 K array was developed, containing a total of 39,724 genes. The remaining genes represented duplicates or sequences failed to meet criteria required for accurate probe design. The data set can be accessed at the Gene Expression Omnibus (GEO) database as platform GPL17440 and series GSE48834.

For functional annotation and GO classification of these genes on this array, similarity search was conducted against the UniProt database (http://www.uniprot.org) and TAIR database (TAIR10_pep_20101214) using BLASTx algorithm with an E value threshold of 10^−5^. Out of 39,724 genes, 26,818 (67.5 %) and 25,238 (63.5 %) showed significant similarity to known proteins in UniProt and TAIR database, respectively. GO functional classification for these sequences was also performed. Additional file 1: Figure S1 summarized the GO functional annotation of the array sequences (Additional file 1: Figure S1). BLAST search and GO classification results showed that the sequences on this array represented a broad range of sweetpotato genes. Collectively, the genes on this array had a broad potential utility for examination of global transcription profiling for diverse tissues at various developmental stages or under a variety of conditions.

### Characterization of sweetpotato root development

**Fig. 1:**
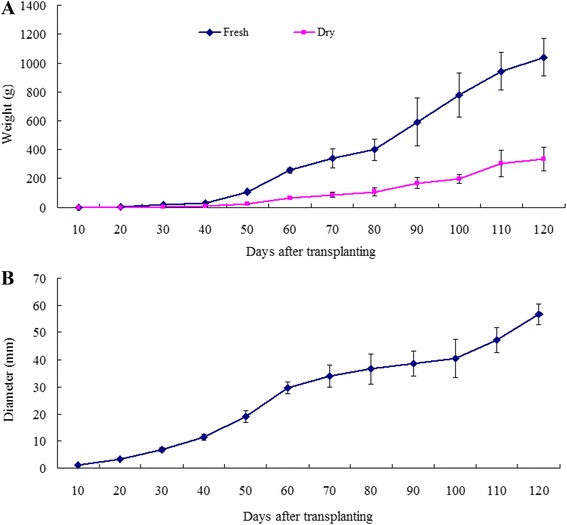
Root growth during sweetpotato root development. **a** Root growth estimated by measurement of fresh weight and dry weight. **b** Root growth estimated by measurement of maximal root diameter. All roots were sampled and measured from one individual sweetpotato plant, and each point is the average of eight plants. SD is denoted by error bars

To create inventories of gene expression at distinct stages in sweetpotato root development, we defined root developmental stages by measuring root fresh weight and dry weight, as well as the maximal root diameter (Fig. 1). At the early stage of root development, fibrous roots are initially formed (root diameter: < 2 mm). As root development continues, some of these fibrous roots become pigmented and begin to thicken, forming the thick roots (diameter: 2–5 mm). Ultimately, some of these thick roots develop into tuberous roots (diameter: >5 mm). Sweetpotato tuberous root formation and development included two phases: the early fibrous and thick root development and the later tuberous root formation and thickening. In order to cover the whole root development, diverse developing roots representing fibrous, thick and tuberous roots at different developmental stages were collected at 10, 15, 20, 30, 60, 90 and 120 days after transplanting (DAT).

### Microarray hybridization and data overview

**Fig. 2:**
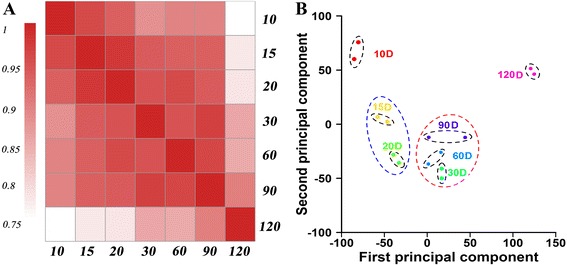
Correlation of gene expression levels between stages and PCA analysis of all arrays. a Correlation of gene expression levels between stages. Each developmental stage is most highly correlated with its adjacent stage. **a** decrease in correlation is observable as the root stage pairs became more distant to each other developmentally. **b** PCA analysis of the seven sweetpotato root developmental stages with two biological replicates. All the two biological replicates of seven samples were excellently assigned together, and four clusters sharing similar expression signatures were identified. D represents days after transplanting

Microarray hybridization experiments were carried out by using mRNAs isolated from representative roots at 10, 15, 20, 30, 60, 90 and 120 DAT with two biological replicates to identify genes that were active during root development. To evaluate the microarray quality, analysis of Pearson correlation coefficients between the two biological replicates were firstly conducted. The results revealed that the Pearson correlation coefficients between the two biological replicates ranged from 0.95 to 0.99, indicating excellent concordance with each other (Additional file 2: Figure S2). The average Pearson correlation coefficients between different stages ranged from 0.95 for 10 and 15 DAT to 0.72 for 10 and 120 DAT samples. In general, Pearson correlation coefficients decreased as the root stage pairs became more distant to each other developmentally (Fig. 2a). For example, the average correlation coefficients between 10 DAT and other samples (15, 20, 30, 120 DAT), were 0.95, 0.92, 0.86, and 0.72, respectively. Interestingly, Pearson correlation coefficients between 30, 60 and 90 DAT samples showed excellent concordance with each other, ranging from 0.94 to 0.96 (Fig. 2a), implying similar global expression trends exist for these root developmental stages.

By applying principal component analysis (PCA) to all 14 arrays, two biological replicates of seven samples were excellently assigned together (Fig. 2b) and further revealed the entire experiment from sample collection, RNA extraction to data extraction was reliable and reproducible. Moreover, distinct transcriptional signatures were also shown in the seven samples by PCA analysis. The developmental stage had a clear influence as the first component, and the overall morphological similarity was also well reflected in PCA distances (Fig. 2b). The first cluster was composed of one time point (10 DAT), representing the fibrous root; the second cluster contained two time points (15, 20 DAT), representing the thick root, an intermediate stage between fibrous root and tuberous root; the third cluster included three time points (30, 60, 90 DAT), representing the tuberous root formation and quick thickening stages; and the last cluster was formed by 120 DAT, representing harvesting time. Consistent with previous report [30], these results clearly demonstrated that developmental stages of root development could be recognized by their transcript expression profiles, which also indicating developing root at a certain stage might have its own distinctive feature of transcriptome.

Hierarchical clustering analysis was also carried out on all the genes and 14 samples, as shown in Additional file 3: Figure S3A. Different mRNA samples were clustered together according to their temporal relationships during root development. As shown by the column dendrogram of the cluster tree, all the two biological replicates clustered together, except the two biological replicates of 90 DAT, one of which was clustered together with 120 DAT samples. Like the PCA distances could reflect the morphological similarity, the column dendrogram of the cluster tree also revealed that the seven mRNA samples were clustered into two sub-trees, corresponding to the early fibrous and thick root development and the later tuberous root formation and quick thickening phases (Additional file 3: Figure S3A).

Taken together, these data showed that (1) the two biological replicates represented excellent concordance with each other, which indicating the experiment was reliable and reproducible; (2) the morphological change of different root development stages could be well reflected by gene expression profiling.

### Genes detected during sweetpotato root development

A stringent protocol was applied to analyze microarray data and restricted our analysis to genes for which the detection call was P (Present) in both biological replicates to reduce the inclusion of false positives. Only probes with consensus detection calls of PP in the two replicates were considered to represent genes detected in any given developmental stage. Probes with discordant detection calls between the two biological replicates [e.g., P and A (absent)] were assigned as insufficient data (INS) and removed from datasets used for further comparative analysis (Methods).

**Fig. 3:**
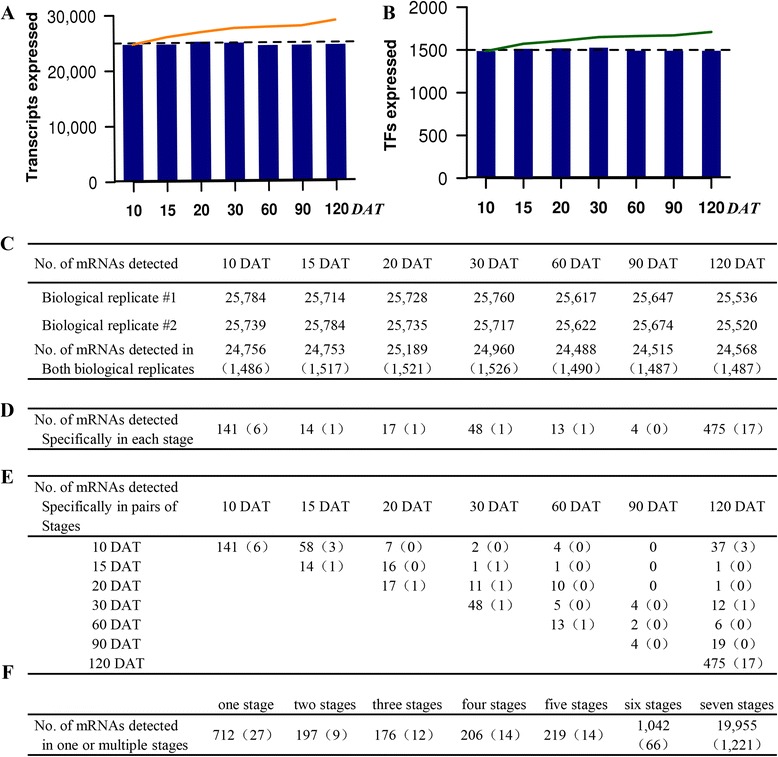
Genes expressed during sweetpotato root development. **a** Transcripts expressed (e.g., P and P in both biological replicates). The bar graphs indicate the number of transcripts expressed in each sample; the lines indicate the cumulative number of expressed transcripts. **b** Transcription factors (TFs) expressed. The bar graphs indicate the number of transcription factors expressed in each sample; the lines indicate the cumulative number of expressed transcription factors. **c** Number of transcripts expressed at each stage of development. Numbers for biological replicates 1 and 2 indicate the number of probes with a detection call of P in each experiment. The number for both biological replicates indicates a consensus probe set detection call of PP. **d**-**f** Number of specific and shared genes expressed at developmental stage. Number in parentheses indicates TFs

At different developmental stages, about 24,000-25,000 genes were identified above the microarray detection limit (Fig. 3a and 3c). In total, 28,964 expressed transcripts (including 1,710 TFs) were cumulatively detected throughout the whole period of root development (Fig. 3a, 3b and 3c). The number of active genes did not vary significantly during the period of root development, ranging from 61 % to 63 % of genes on the array. The proportion of TF transcripts relative to total genes within a population was the same for all stages (i.e., ≈6 %). To determine the spectrum of TFs during root development, TFs detected in each developmental stage were organized into major TF families. In total, 77 TF families were identified, and all major TF families were represented at each developmental stage (Table S1 in Additional file 4). The similar number of active gene found in each sample reflected a large overlap in transcripts even in very distinct development stages. Like the similar number of active genes in each sample, expression dynamics of different stages could not be easily distinguished from each other (Additional file 5: Figure S4). In general, relative expression levels of genes in sweetpotato roots were shifted towards lower values, with few expressed above average levels.

Taken together, these data showed that (1) the number of genes at different developmental stages did not vary significantly during the period of root development; (2) at least 28,000 genes (including at least 1,700 TFs) were active throughout sweetpotato root development, and (3) all major TF families were represented at each developmental stage.

### Specific genes detected during sweetpotato root development

A small number of genes were detected specifically to each stage at the level of the microarray, including those encoding TFs (Fig. 3d and Table S2 and S3 in Additional file 4). The stage-specific genes included a range of functional categories, although almost half of them encoded predicted or unknown proteins (Table S2 and S3 in Additional file 4). A total of 712 (including 27 TFs) genes were specifically detected in individual stages. Among them, 141, 48 and 475 stage-specific genes, including 6, 1, 17 TFs were, respectively, observed at 10, 30 and 120 DAT when important differentiation and morphological events occurred during root development (Fig. 3d and Table S2 and S3 in Additional file 4). The 10 DAT-specific functional genes included those encoding fatty acid hydroxylase superfamily (IBTC1038130 and IBTC1047671), and auxin-responsive GH3 family protein (IBTC1040253 and IBTC1044372). Importantly, the 10 DAT-specific TFs included those encoding DA1-related protein 2 (IBTC1071835), AGAMOUS-like 20 (IBTC1056780), myb domain protein 84 (IBTC1056100), Auxin-responsive protein IAA7 and IAA16 (IBTC1040064, IBTC1028248, IBTC1025877). DA1-related protein 2 has recently been shown to control root meristem size [31]. By contrast, only 48 genes (including 4 TFs) were specific in the remaining four stages (Fig. 3d and Table S2 and S3 in Additional file 4).

Then we compared the genes detected in multiple developmental stages to determine whether there were root-period specific genes in addition to those unique to individual stage (Fig. 3e-f). We observed that pairs of root stages that were close to each other developmentally (e.g., 10 and 15 DAT, 15 and 20 DAT) had small sets of genes that were not detected at other developmental stages at the level of the microarray (Fig. 3e and Table S4 and S5 in Additional file 4). For example, the 10 and 15 DAT samples had 58 specific genes (including 3 TFs) that were not detected in other stages. Similarly, the 15 and 20 DAT samples had 16 genes that were not detected at any other stages investigated. By contrast, there were not any detectable pair-specific genes neither between 10 and 90 DAT samples nor between 15 and 90 DAT (Fig. 3e and Table S4 and S5 in Additional file 4). Additionally, a total of 1,643 genes (including 106 TFs) were identified to express in three to six stages (Fig. 3f, Table S4 and S5 in Additional file 4).

Analysis of GO terms enriched in both the root-stage specific and root-period-specific genes was listed in Table S6 and S7 in Additional file 4. Especially, GO enrichment analysis of both the root-stage-specific and root-period-specific TFs indicated that in early fibrous and thick root development stages (i.e. 10, 15 and 20 DAT), TFs were enriched in sequences encoding SHORT-ROOT (SHR) (IBTC1062233), NAC domain containing protein 6 (IBTC1014629), WRKY22 (IBTC1066366) and WRKY27 (IBTC1073827). The *SHORT-ROOT* gene was already confirmed controlling radial patterning of the *Arabidopsis* root through radial signaling [32]. Whereas the 30, 60 DAT and latter stages, TFs included those encoding ABA-responsive element binding protein 3 (IBTC1010741), Homeodomain-like superfamily protein (IBTC1015565), BEL1-like homeodomain 1 (IBTC1062736) (Table S7 in Additional file 4). These different regulatory genes were probably involved in the tuberous root expansion. Taken together, consistent with the substantial overlap in expressed genes between samples, there were only a few specific genes, including those encoding TFs, for each stage and period of root development at the level of this microarray.

### Shared genes detected during sweetpotato root development

In contrast with the few root-stage and root-period-specific genes (Fig. 3d-f), 19,955 genes (including 1,221 TFs) were shared expressed during root development (Fig. 3f), indicating that most diverse root genes were active across entire root development. Using the 10 DAT sample as a reference, 26.9 % of shared expressed genes (5,368, including 435 TFs) changed by at least 2-fold in at least one developmental period at the cut-off *P*-value < 0.05. Such 5,368 shared expressed genes were defined as differentially expressed genes in this study.

**Fig. 4:**
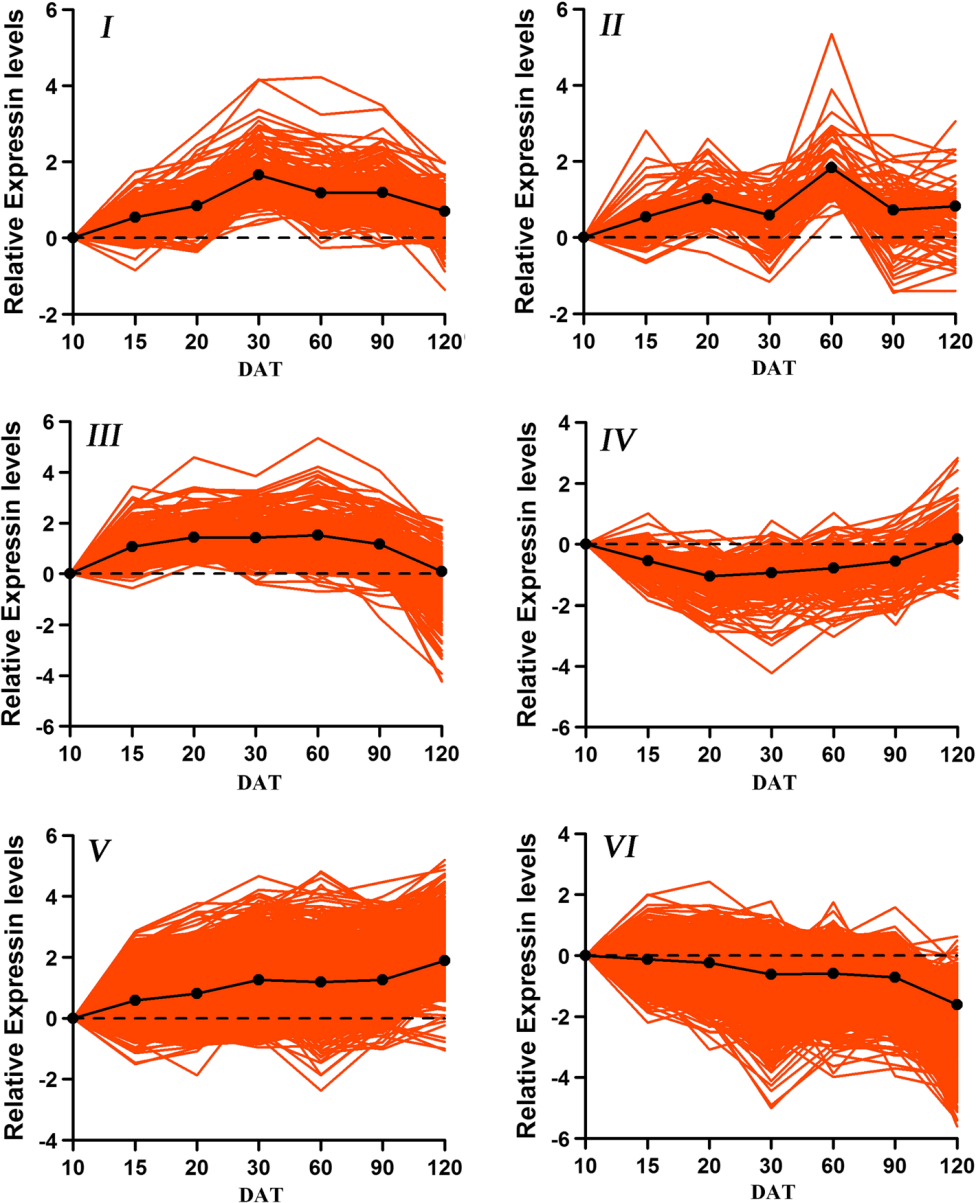
Clusters of differentially expressed genes. We identified six prominent clusters of genes with similar expression dynamics. Expression levels across development for genes in each cluster were indicated by colored lines, and the thick black lines represented the average gene accumulation pattern for all genes in each cluster

To cluster the genes showing similar expression profiles during root development, hierarchical clustering analysis was carried out on the differentially expressed genes (Additional file 3: Figure S3B). We identified 6 prominent gene clusters. The cluster I and cluster II were up-regulated at 30 DAT and 60 DAT, respectively. The cluster III was up-regulated between 15 to 90 DAT. The cluster IV was down-regulated at 20 DAT. The cluster V and cluster VI were monotonically increasing or decreasing during root development (Additional file 3: Figure S3B and Fig. 4). Among of these differentially expressed genes, 19.0 % and 4.8 % of them changed more than 5-fold and 10-fold, respectively, and the highest gene abundance change for the expressed genes was almost 100-fold (gene encoding WRKY transcription factor).

GO analysis of clustered genes revealed enrichment for genes programming different processes at specific stages of root development (Table S8 in Additional file 4). For example, the genes active in cluster V were enriched for information involved in auxin mediated signaling, sugar signaling, abscisic acid signaling, protein amino acid dephosphorylation, thylakoid membrane organization and biogenesis, chloroplast organization and biogenesis, chlorophyll biosynthesis, glycogen synthesis, starch synthesis, amylopectin biosynthesis and carotene biosynthesis, and the cluster VI were enriched in oxidation reduction, protein amino acid phosphorylation, response to cadmium ion and calcium ion transport, regulation of stomatal movement, lipid catabolism, coumarin biosynthesis, fatty acid biosynthesis, lignin biosynthesis. These GO terms reflected major physiological events of fibrous root elongation, tuberous root initiation and expansion. For example, lignin biosynthesis and fatty acid biosynthesis in fibrous root elongation, and starch synthesis in later tuberous root thickening [28, 33]. Each cluster contained TFs that may be important for regulating the GO-term biological processes that occurred during the corresponding developmental period (Table S9 in Additional file 4). For example, WRKY DNA-binding protein 75 (IBTC1018518), RAV transcription factor (IBTC1059736), ARF7 (IBTC1074823), ARF16 (IBTC1063423), were active in cluster VI. These TFs have been shown to modulate/control root development and phosphate acquisition [34], shoot regeneration and photoperiodicity [35], lateral root formation [36], root cap formation [37]. In cluster V, MADS-box transcription factor family protein (IBTC1007376), CCT motif family protein (IBTC1018451), CCCH-type zinc finger family protein (IBTC1027692), Dof zinc finger protein (IBTC1002667), BEL1-like transcription factor (IBTC1014968), Class-I knotted1-like homeobox protein were prevalent and involved in initial thickening growth of storage root of sweetpotato [23], protein import and synthesis in leaf chloroplasts [38], the regulation of rice plant architecture [39], modulating the carbohydrate metabolism in the storage roots of sweetpotato [40], affecting secondary metabolism [41], regulating tuber formation and many aspects of vegetative development [42, 43], controlling cytokinin levels in the sweetpotato storage roots [21]. Meanwhile, the KEGG pathway enrichment analysis was also carried out for these clustered genes (Table S10 in Additional file 4). Taken together, these results showed that (i) most root genes, including TFs were shared expressed during root development, (ii) shared expressed root genes underwent significant quantitative changes and these differentially expressed genes were grouped into six prominent clusters, and (iii) genes within each cluster encoded proteins involved in important root developmental biological processes.

### Verification of gene expression patterns by RT-PCR

**Fig. 5:**
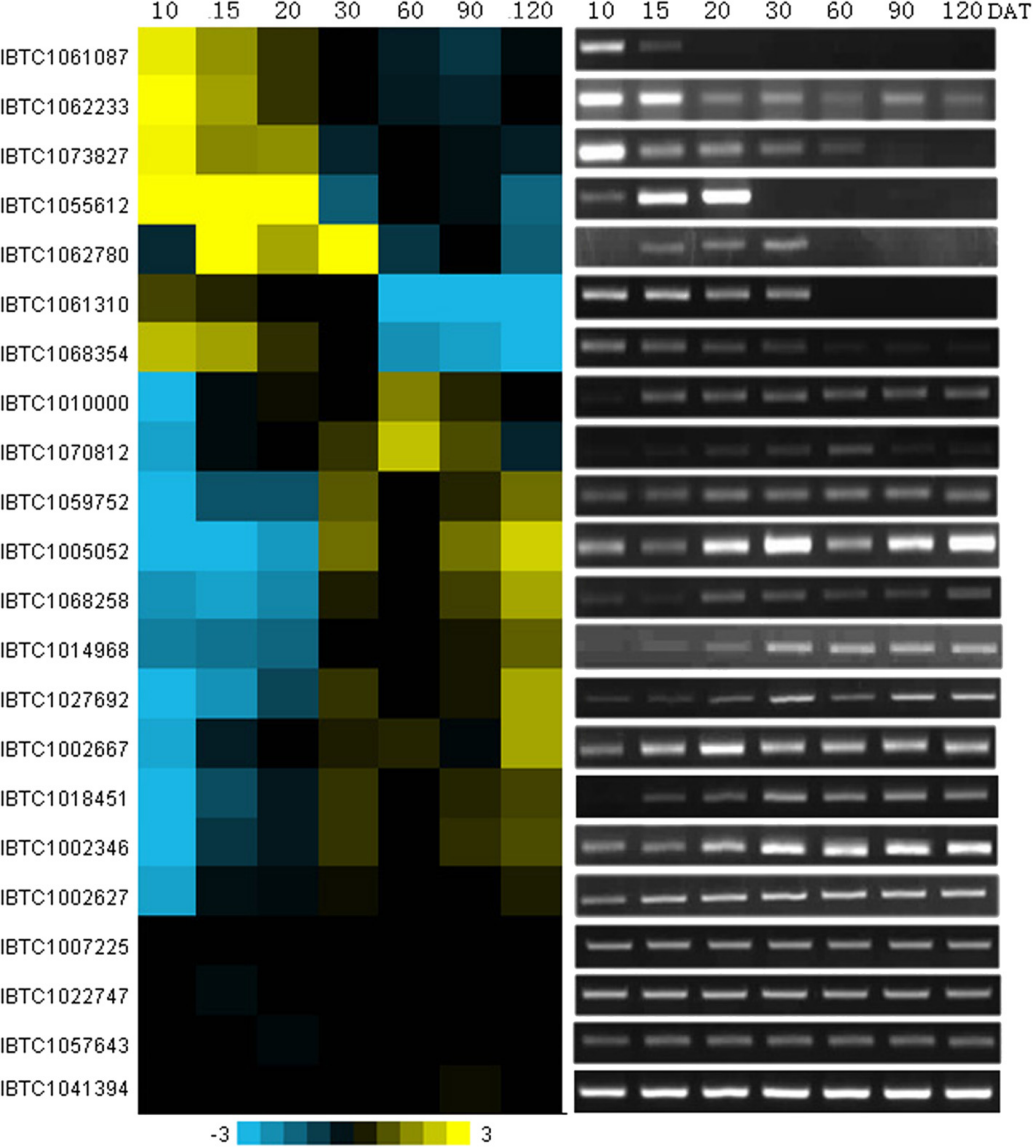
Comparison of gene expression patterns between microarray hybridization data and RT-PCR. For microarray data verification, RT-PCR analysis was performed on 22 selected genes, including specific, differentially expressed and constitutively expressed genes. DAT represented sweetpotato root developmental stages (days after transplanting)

To validate the microarray data, RT-PCR was performed using the RNA extracted from the three biological replicates at different developmental stages that were used in this microarray analysis. A total of 22 expressed genes, including 14 TFs were selected for verification (Table S11 in Additional file 4). For 12 tested specific genes, half of them, however, were also detected in one or more other stages at greatly reduced levels, which indicating that this type of specific genes could also be detected at other stages, but probably below the detection limit of our microarray experiments. This similar result was also reported by Brandon H. Le *et al.* [44]. Six differentially expressed and 4 constitutively expressed genes showed excellent consistence with the microarray data (Fig. 5). Taken together, these results showed that expression profiling of most tested genes were consistent with the microarray data, but some of the specific genes were active not only in target stage (s), but also in other stage (s) with greatly reduced level.

## Discussion

In this study, a 60-mer microarray representing 39,724 genes were designed and utilized for characterizing and profiling gene expression patterns during root development to uncover candidate genes and key transcriptional regulators relating to tuberous root initiation and development in sweetpotato, a species without a reference genome. Pearson correlation coefficient, PCA as well as hierarchical cluster analyses revealed that the two biological replicates used in this experiment showed high concordance with each other, which indicating the entire experiment was reliable and reproducible. In order to reduce the possible inclusion of false positives, a stringent protocol was also used to analyze this microarray data. The numbers of genes detected at each stage of development were calculated from probes with only consensus detection calls of PP in the two replicates. At different developmental stages, about 24,000-25,000 genes were identified above the microarray detection limit (Fig. 3c). In total, 28,964 expressed transcripts were cumulatively detected throughout the used seven stages of root development. Furthermore, to identify specific and shared expressed genes during the seven stages of root development, any probes with consensus detection calls of INS between the two replicates in at least one developmental stage were also removed from all sample datasets. In total, 8,275 (20.8 %) INS probes and 8,942 (22.5 %) AA probes (detection calls between the two biological replicates were AA in all developmental stages) were detected and removed. Thus, in this paper, we can also assume that at least 29,000 genes were needed to orchestrate the complete sweetpotato root development, and the detected specific and shared expressed genes represented the minimum number of genes that were active during root development.

In this study, most genes were shared expressed across different developmental stages, although significant quantitative changes occurred in individual gene abundance that corresponding with specific developmental stages and/or periods. In total, we detected 5,368 differentially expressed genes (including 435 TFs) across all developmental stages. GO and KEGG pathway enrichment analysis showed categories and pathways involved in sugar signaling, abscisic acid signaling, protein amino acid dephosphorylation and starch synthesis were up-regulated and enriched at later tuberous root expansion stage, whereas protein amino acid phosphorylation, lignin biosynthesis, coumarin biosynthesis, fatty acid biosynthesis and auxin signaling were highly active during the early stage of fibrous and thick root development and then down-regulated later. In potato, sugars were thought to act as the driving force behind the formation and growth of the sink tuber as sucrose was the main photoassimilate transported from the leaves towards the expanding sink organ [45]. During the rapid tuber growth phase, the active sink accumulated large amounts of storage compounds, mainly in the form of starch [46]. In sweetpotato, previous studies showed that cytokinin and auxin levels have been found to be high during the early tuberous root formation [15, 19, 23]. The later stage of tuberous root development was positively correlated with concentrations of abscisic acid and cytokinin, but not with IAA levels [19]. So it was not surprising that in the later tuberous root thickening stage, sugar signaling, abscisic acid signaling and starch synthesis were prevalent. During the early fibrous and thick root development, two processes were involved, including the fibrous root elongation and the cessation of the elongation to radial growth. Qin *et al.* reported that saturated very-long-chain fatty acids could promote cotton fiber and *Arabidopsis* cell elongation by activating ethylene biosynthesis [33]. Our expression results were consistent with all these reports, which indicated that the detected differentially expressed genes, including those encoding TFs, during root development would be of great value in uncovering molecular mechanism relating to tuberous root initiation and development. In addition, tuberous root of sweetpotato is composed of about 70 % of starch. This required not only the synthesis and deposition of a large amount of starch but also the degradation or clearance of other metabolites. Data gathered from our transcript profiles demonstrated the dynamic changes of metabolism network centering on starch synthesis during tuberous root thickening stage. While starch synthesis was prevailing, many metabolism pathways that were active during the early fibrous and thick root development were repressed. For example, fatty acid, coumarin and flavonoid synthesis were down-regulated. Thus, in the tuberous root thickening stage, metabolic pathways were coordinated to direct carbon flux into starch. This type of metabolism regulation is common to many crop species, such as cellulose in cotton fiber, fatty acid in oilseeds and starch in cereal grains. In cotton, mature fiber is composed of nearly pure cellulose, and genes involved in cellulose synthesis accumulate largely during secondary cell wall synthesis [47]. A large amount of oil bodies are accumulated in later developing oilseed rape embryos, but starch is degraded. More interestingly, sucrose and hexose are also found to be mobilized for fatty acid synthesis via the oxidative pentose phosphate pathway [48, 49].

By contrast, only a small set of genes, including those that encode TFs, were detected specifically at each root developmental stage. Interestingly, more numbers of root-stage-specific genes were observed at the stages of 10 DAT and 30 DAT when major morphological events occurred during root development. As shown in Fig. 1, the average root diameter estimated by measurement of maximal root diameter at 10, 15, 20, 30 DAT were 0.96 mm, 2.12 mm, 3.36 mm, and 6.85 mm, respectively. Roots sampled at 10 DAT, 15 and 20 DAT, 30 DAT could be respectively defined as fibrous roots, thick roots and tuberous roots according to adventitious roots classification in sweetpotato [fibrous roots (<2 mm), thick roots (2–5 mm) and storage roots (>5 mm)] [3, 5, 20]. Actually, the thick root was a transitional stage from fibrous root to tuberous roots. Surprisingly, the largest numbers of root-stage-specific genes were observed in the 120 days. In addition to a small set of root-stage specific genes, more than 1,800 genes, including 115 TFs, were observed in mosaic combinations of two to six stages, although the number was significantly less than those genes shared across development (Fig. 3e-f and Table S2-5 in Additional file 4). Among these period-specific genes, 77.2 % of them accumulated within temporally contiguous periods that corresponded with important root developmental events as well, for example, 10–15 DAT, 10-15-20 DAT and 30-60-90-120 DAT. Most of genes detected specifically in two and three stages were respectively 10–15 DAT and 10-15-20 DAT, key times that were required for thick root and tuberous root formation (Fig. 1 and Table S12 in Additional file 4).

We identified 142 stage- and period-specific TFs that most likely would play important roles in regulating root development. The functions of most of these TFs identified here were not known in sweetpotato, however, these TFs were enriched for known regulators of root gravitropism, cell division and differentiation, hormone-mediated signaling during root development, and several of them have been confirmed involving in the early root development [e.g., DA1-related protein controlling root meristem size [31], SHORT-ROOT controlling root radial patterning formation [30], and secondary tuberous root formation and development [e.g., MADS-box transcription factor involving in initial thickening growth of storage root of sweetpotato [23, 24], BEL1-like transcription factor for regulating tuber formation in potato [41, 42]. All the results strongly suggest that the remaining specific TFs would also play critical regulatory roles during root development. The critical question is what roles the remaining TFs in our dataset play in root development.

## Conclusions

In conclusion, whole-transcritome gene expression during the process of sweetpotato root development was characterized using the newly designed sweetpotato microarray, and specific and differentially expressed genes, including those encode TFs, were identified and analyzed in detail. At the present time, in sweetpotato, the roles of most regulatory genes in controlling tuberous root initiation and development and how root genes are organized into regulatory networks remain largely unknown. The specific and differentially expression genes (including TFs) identified in our study should provide an important starting point for understanding how gene activity is coordinated for programming tuberous root formation and development.

## Methods

### Plant materials

Stem cuttings of sweetpotato (*Ipomoea batatas*. cv. Guangshu 87) were grown in the field from August to November in 2011 at the experimental station of Guangdong Academy of Agricultural Sciences (GAAS). Developing roots were collected precisely at 5-day interval during the early 60 DAT and then 10 days interval until harvesting time. The maximal root diameter was measured using a vernier caliper. Fresh and dry weight were also measured at each collecting stage using descriptors and data standard for sweetpotato [50]. For microarray analysis, fibrous roots (10 DAT), thick roots (15 and 20 DAT) and tuberous roots (30, 60, 90, 120 DAT) at different developmental stages were used. All the samples were immediately frozen in liquid nitrogen after collecting, and stored at −80 °C prior for total RNA extraction.

### Sweetpotato oligonucleotide microarray construction

The microarray design was based on the sequences including 66,418 ESTs (31,685 contigs and 34,733 singletons) from sweetpotato gene index established by Schafleitner *et al.* [27], 56,516 developed by Wang *et al.* and 58,681 generated in house [2, 51]. These ESTs were assembled using the TIGR Gene Indices Clustering Tools (TGICL) [29], and 87,492 potential unique ESTs were generated. A total of 71,999 in situ synthesized 60-mer oligonucleotide probes representing 39,724 sweetpotato genes were constructed on the microarray using Roche NimbleGen’s photo-mediated synthesis chemistry with Maskless Array Synthesizer (MAS) system. For functional annotation and GO classfication of these sequences on this array, similarity search was conducted against the UniProt database (http://www.uniprot.org) and TAIR database (TAIR10_pep_20101214) using BLASTx algorithm with an E value threshold of 10^−5^. Blast2GO program [52] was used to get GO annotation according to molecular function, biological process and cellular component ontologies (http://www.geneontology.org).

### Hybridization and data extraction

Total RNA was isolated using the RNeasy Plant Mini Kit (Qiagen, Shanghai, China). RNA quality and quantity were determined using a NanoDrop ND-1000 Spectrophotometer (Nanodrop Technologies, Wilmington, DE) and verified for degradation using a 2100 Bioanalyser RNA Nanochip (Agilent, Palo Alto, CA). RNA labeling was carried out using CapitalBio cRNA Amplification and Labeling Kit following the manufacturer’s protocols. Arrays were hybridized at 42 °C for 16 h using NimbleGen Hybridization System 12 and then washed three times. Hybridized microarray slides were scanned with Nimblegen MS 200 Microarray Scanner, and images were saved in Tagged Image File Format files (TIFF, .tif). The signal intensities of all spots on each image were quantified by using NimbleScan v2.5, and then further normalized using RMA (Robust Multi-Array Analysis). Microarray representing biological replicates were hybridized, washed, and scanned at the same time to minimize variability. All original files and processed data were deposited in the Gene Expression Omnibus (GEO) database (www.ncbi.nlm.nih.gov/geo).

### Data analysis of microarray hybridization

In order to get more reliable data and reduce the inclusion of false positives, a stringent protocol was applied to define and analyze microarray data. Probes with signal values above our microarray detection limit were given a detection call “present” (P), and signal values below the detection limit were designed as "absent" (A). Each probe was manually assigned a consensus detection call in Microsoft Excel based on the detection calls of both biological replicates of an RNA sample. Probes with signal detection calls of P or A in both biological replicates were assigned consensus detection calls of PP and AA, respectively. In general, the detection calls for biological replicates agreed with one another ≈ 95 % of the time, on average, with a range of 94–97 %. We also calculated cumulative present calls by counting the samples in which a given probe had been called present. By contrast, probes with discordant detection calls for the two biological replicates (e.g., P and A) were assigned a consensus detection call of Insufficient (INS). On average, ≈4.7 % of the probes were assigned a consensus detection call of INS for a given pair of biological replicates, with a range of 2.7–5.7 %. These percentages were less than the discordance values reported by others [44, 53].

### Filtering of microarray data

Because of the uncertainty in INS calls, only probes with detection calls of PP (i.e., P in both biological replicates) were considered to represent a gene detected in any given developmental stage. Microsoft Excel was used to remove probes with calls of INS to compare gene activity between different developmental stages. For the analysis of gene activity during root development, we removed 8,275 probes with consensus detection calls of INS in at least one developmental stage from all sample datasets. An additional 8,942 probes with consensus detection calls of AA across all developmental stages were also removed, leaving 22,507 probes passing filters (57 % of the microarray probe sets).

Identification of specific and shared expressed genes was performed according to Brandon *et al.* [44]. To identify root-stage specific genes, Microsoft Excel was used to filter probes with a detection call of PP in one sample and AA across all other samples. Likewise, to identify multiple-stage-specific gene sets, we filtered for probes with a detection call of PP in two or more stages and AA in the remaining stages. To identify genes shared by all developmental stages being analyzed, we filtered for probes with a detection call of PP in all biological samples being compared. Shared expressed genes were classified into constitutively expressed genes and differentially expressed genes. Using the 10 DAT sample as a reference, the differentially expressed genes were determined by calculating *P*-values from one way ANOVA and fold changes between each comparison for each gene with a selection threshold of fold change ≥ 2.0 and *P*-value <0.05 [54].

### Bioinformatics analysis of microarray data

Z scores calculation. Expression values of probe sets for each sample were converted into Z scores using a two-step process. First, we averaged expression values for each sample, and from these numbers, we determined a single mean (μ_G_) and standard deviation (σ_G_) for calculation of gene expression Z scores. The Z score for the *i*^th^ gene in the *j*^th^ sample is given by the equation z_*ij*_ = (x_*ij*_-μ_G_)/σ_G_. For the Z score of sweetpotato root, expression values for all samples were averaged for calculation.

Hierarchical Clustering. All the genes on this chip and the identified differentially expressed genes were used for hierarchical clustering with the average linkage method, respectively, and the cluster data were visualized by the Treeview program [55].

PCA. Principal Component Analysis (PCA) was performed to reduce dimensions from all the gene values to three dimensions for all seven samples with two biological replicates by using a standard-PCA algorithm.

Gene Ontology and KEGG pathway enrichment analysis. The specific genes and differentially expressed genes in each cluster were evaluated for enrichment of biological functions in GO categories and biochemical pathways by use of the CapitalBio Molecule Annotation System (http://bioinfo.capitalbio.com/mas3/). Only GO terms and pathways with a *P* < 0.01 are listed in Tables S6-10 Additional file 4.

### RT-PCR analysis

First-strand cDNA was synthesized from 1 μg total RNA using the Superscript first-strand synthesis system for RT-PCR (Invitrogen). Gene-specific RT-PCR primers were designed with Primer 5.0 and synthesized commercially (Invitrogen) as listed in Additional file 4: Table S11. The PCR reactions were performed in a 20 μl reaction volume containing a 2× Taq Master Mix, 50 ng cDNA, 400 nM of forward primer, and 400 nM of reverse primer in a Bio-Rad thermocycler. The RT-PCR cycles were as follows: initiation with a 5-min denaturation at 95 °C, followed by 30 cycles of amplification 10 s of denaturation at 95 °C, 20 s of annealing at 55–58 °C, 30 s of extension at 72 °C. After a final extension at 72 °C for 10 min, and 10 μl PCR product were used for running gel. All of the samples were measured in triplicate.

### Availability of supporting data

The microarray data set are available at the Gene Expression Omnibus (GEO) database as series GSE48834. http://www.ncbi.nlm.nih.gov/geo/query/acc.cgi?acc=GSE48834.

## Additional files

Additional file 1: Figure S1. Gene Ontology classification of sequences on this array. The results are summarized in three main categories: Biological process, Cellular component and Molecular function. Additional file 2: Figure S2. Correlation of microarray data between biological replicates. The microarray signal intensities from two biological replicates were plotted on X-Y scatter plots. All probe sets were plotted. The Pearson correlation coefficient (r) for each pair of RNA samples was calculated using Microsoft Excel. Additional file 3: Figure S3. Clustering of microarray samples and genes during sweetpotato root development. (A) Hierarchical clustering of all microarray samples and genes was carried out by using Cluster 3.0. The signals are shown in a blue - yellow color scale, where blue represents lower expression and yellow represents higher expression. (B) Only the differentially expressed genes were included in the clustering analysis. Six prominent individual clusters were shown using roman numerals. Additional file 4: Table S1-12.
**Table S1.** Identification of transcription factor families in root mRNA populations at specific stages of root development. **Table S2.** Stage-specific mRNAs detected by the microarray during rooot development. **Table S3.** Stage-specific TFs detected by the microarray during rooot development. **Table S4.** Multiple stage-specific mRNAs detected by the microarray during root development. **Table S5.** Period-specific TFs detected by the microarray during rooot development. **Table S6.** GO terms enriched in stage-specific and period-specific mRNAs. **Table S7.** GO terms enriched in stage-specific and period-specific TF mRNAs. **Table S8.** GO terms enriched in mRNA clusters identified by hierarchical clustering analysis of differentially expressed mRNAs. **Table S9.** GO terms enriched in mRNA clusters identified by hierarchical clustering analysis of shared differentially expressed TF mRNAs. **Table S10.** KEGG pathways enriched in mRNA clusters identified by hierarchical clustering analysis of differentiallyexpressed mRNAs. **Table S11.** Selected genes and primers used for validation of microarray-based gene expression by RT-PCR. **Table S12.** Statistics of multiple stage-specific mRNAs in contiguous and non-contiguous periods. Additional file 5: Figure S4. Histograms of relative expression levels (Z scores) of different samples.

## Abbreviations

TF: Transcription factor
DAT: Days after transplanting
GO: Gene Ontology
KEGG: Kyoto Encyclopedia of Genes and Genomes
PCA: Principal component analysis
NGS: Next generation sequencing
