# Supplementary figures and images for: Temporal patterns of gene expression associated with tuberous root formation and development in sweetpotato (Ipomoea batatas)
Source: BMC Plant Biol. 2015 Jul 16;15:180. doi: 10.1186/s12870-015-0567-5 (PMC4502468; doi:10.1186/s12870-015-0567-5)

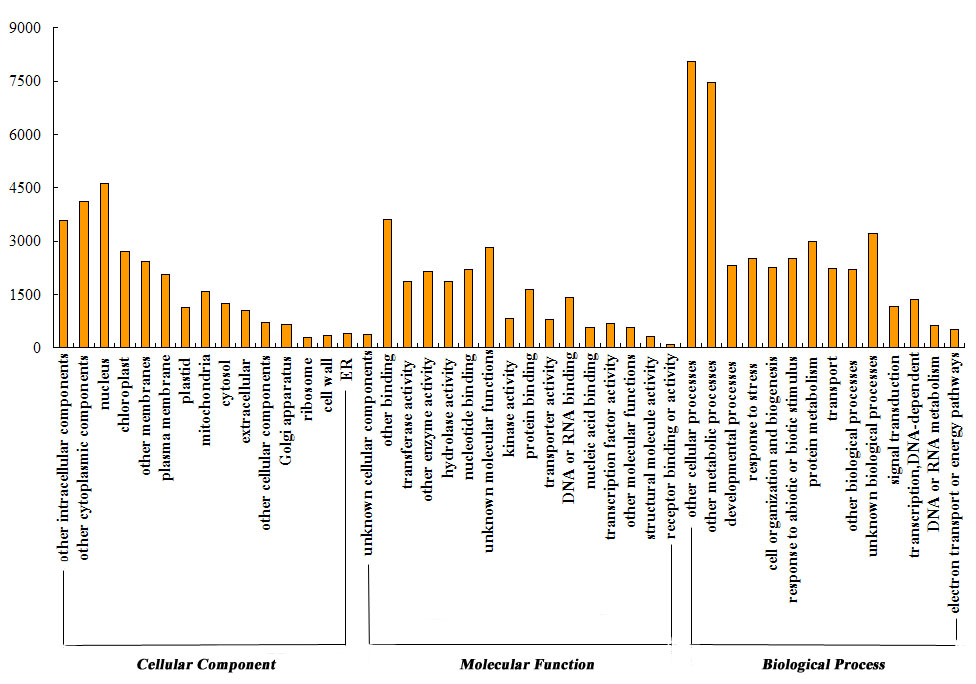

Supplement: Additional file 1: Figure S1. — Gene Ontology classification of sequences on this array. The results are summarized in three main categories: Biological process, Cellular component and Molecular function. [file 12870_2015_567_MOESM1_ESM.jpg]

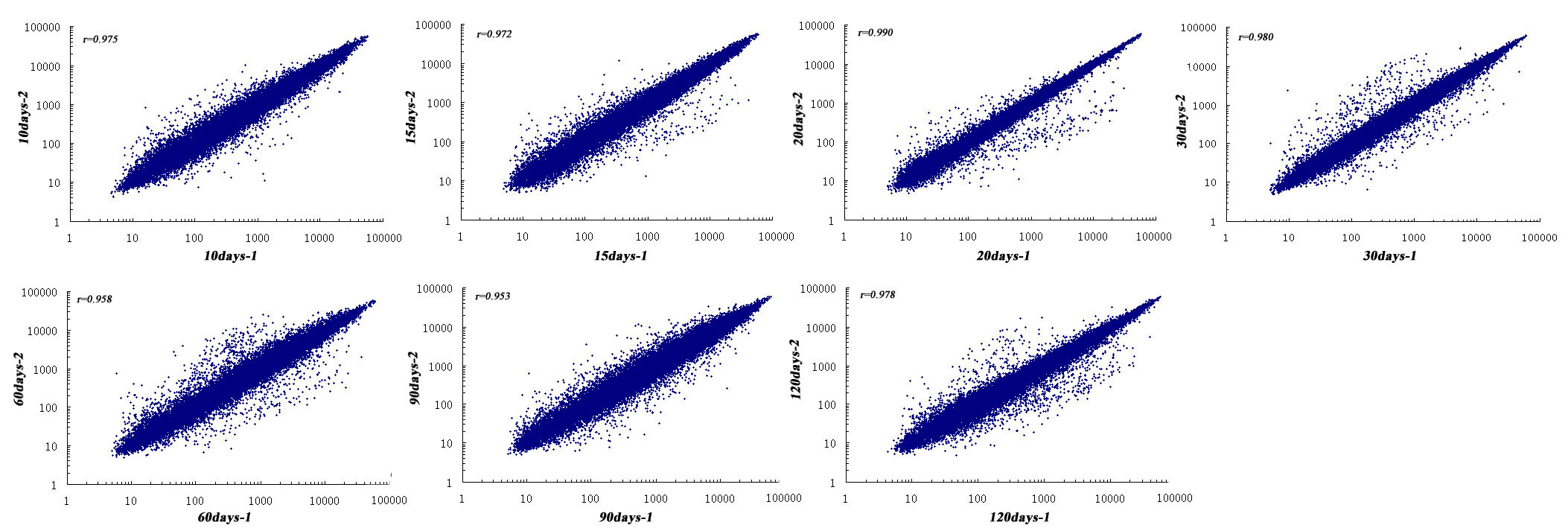

Supplement: Additional file 2: Figure S2. — Correlation of microarray data between biological replicates. The microarray signal intensities from two biological replicates were plotted on X-Y scatter plots. All probe sets were plotted. The Pearson correlation coefficient (r) for each pair of RNA samples was calculated using Microsoft Excel. [file 12870_2015_567_MOESM2_ESM.jpg]

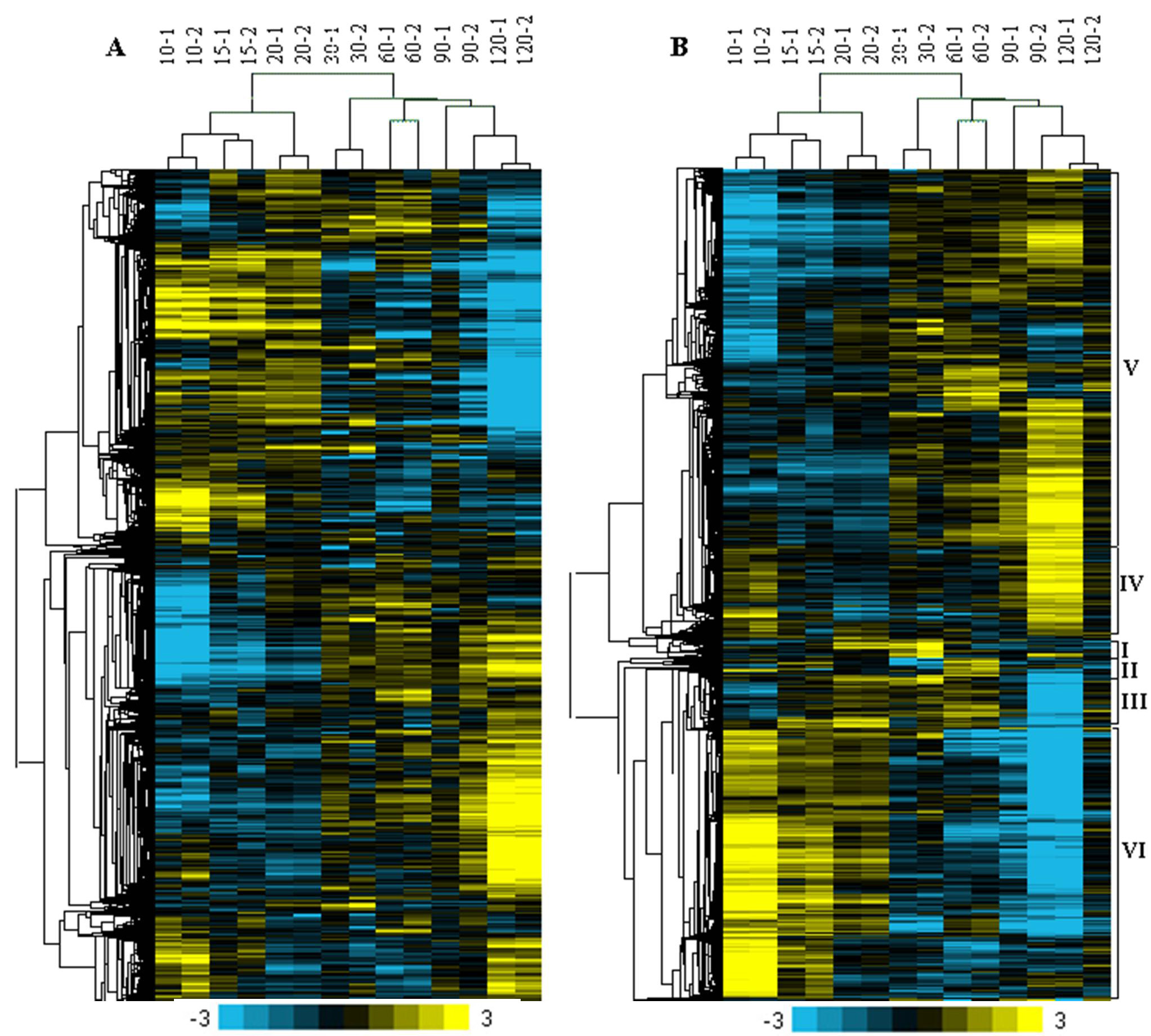

Supplement: Additional file 3: Figure S3. — Clustering of microarray samples and genes during sweetpotato root development. (A) Hierarchical clustering of all microarray samples and genes was carried out by using Cluster 3.0. The signals are shown in a blue - yellow color scale, where blue represents lower expression and yellow represents higher expression. (B) Only the differentially expressed genes were included in the clustering analysis. Six prominent individual clusters were shown using roman numerals. [file 12870_2015_567_MOESM3_ESM.jpg]

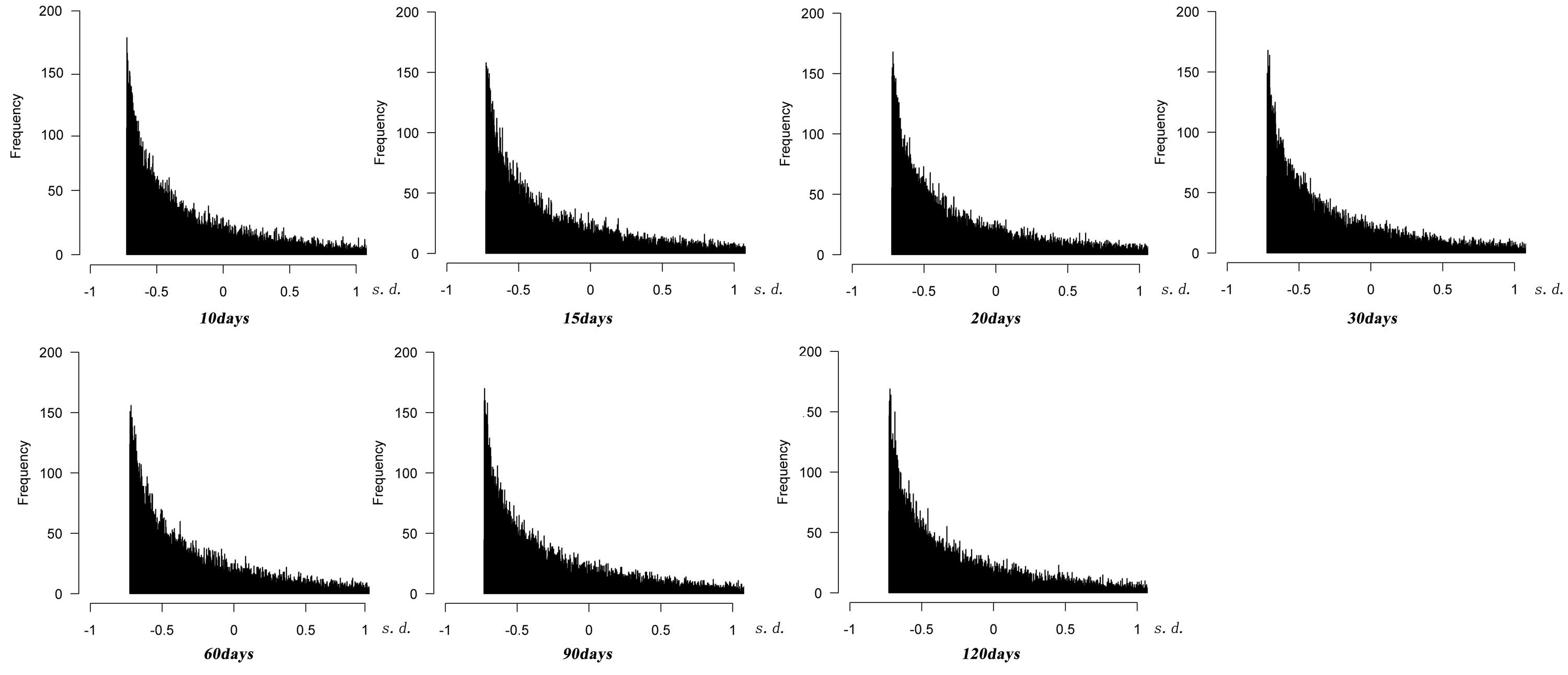

Supplement: Additional file 5: Figure S4. — Histograms of relative expression levels (Z scores) of different samples. [file 12870_2015_567_MOESM5_ESM.jpg]
